# Medicinal Plants Used in Wound Care: A Study of *Prosopis africana* (Fabaceae) Stem Bark

**DOI:** 10.4103/0250-474X.70479

**Published:** 2010

**Authors:** A. C. Ezike, P. A. Akah, C. O. Okoli, S. Udegbunam, N. Okwume, C. Okeke, O. Iloani

**Affiliations:** Department of Pharmacology and Toxicology, Faculty of Pharmaceutical Sciences, University of Nigeria, Nsukka, Enugu State - 410001, Nigeria; 1 Department of Veterinary Surgery, Faculty of Veterinary Medicine, University of Nigeria, Nsukka, Enugu State - 410001, Nigeria

**Keywords:** Haemostatic activity, antibacterial activity, *Prosopis africana*, wound care

## Abstract

The effects of the methanol extract of the stem bark of *Prosopis africana* (Guill., Perrott. and Rich.) Taubert (Fabaceae) on bleeding/clotting and coagulation time, excision and dead space wounds were studied in rats. Also, the extract was subjected to antibacterial, and acute toxicity and lethality (LD_50_) tests. The extract significantly (*P*<0.05) reduced bleeding/clotting and coagulation time in rats. It also reduced epithelialization period of excision wounds in rats and inhibited the growth of laboratory strains of *Staphylococcus aureus, Bacillus subtilis, Salmonella typhi, Pseudomonas aeruginosa* and *Klebsiella pneumoniae* to varying extents. Acute toxicity and lethality (LD_50_) test on the extract established an LD_50_ of 774 mg/kg (i.p) in mice while phytochemical analysis gave positive reactions for alkaloids, saponins, tannins, flavonoids, steroids, terpenoids and carbohydrates. The results of this study demonstrate the beneficial effects of the stem bark of *P. africana* in wound care.

Wound healing is a complex and dynamic process involving a highly regulated cascade of biochemical and cellular events designed to restore tissue integrity following injury[1,2]. These events do not occur in series, but rather partially overlap in time[3,4]. Injury to tissues immediately results to bleeding (which may be life-threatening depending on the severity), with subsequent activation of the inflammatory response and regeneration of epithelial tissues. The injury and associated acute inflammatory response lead to necrosis of specialized cells and damage to the surrounding matrix[5]. Consequently, the host tissue must activate the healing process to replace dead tissues with healthy ones[6]. Classically, wound healing is thought to occur in three major phases: thrombosis and inflammation, proliferation (comprising angiogenesis, granulation tissue formation, collagen production, epithelialization and wound contraction), and tissue remodeling and maturation (scar formation). The inflammatory phase prepares the area for healing, the proliferative phase rebuilds the structure and the differentiation phase provides the final form[7]. These biological processes culminate in the replacement of normal skin structures with fibroblastic mediated scar tissue[1].

*Prosopis africana* (Guill., Perrott. and Rich.) Taubert (Fabaceae) is one of the plants used to stimulate wound healing in traditional medicine of south-eastern Nigeria. It is a tree with a very hard wood[8] and easily distinguishable by its dark rough bark, pale drooping foliage with small pointed leaflets and sausage-shaped fruit. The morphological features have been described in detail[8,9]. In Nigeria, *P. africana* is variously called *Okpei* (Igbo), *Ayan* (Yoruba), *Okpeghe* (Idoma and Tiv), and *Kiriya or Kiriaya* (Hausa). A poultice of the boiled seeds is usually applied externally to relieve sore throat, while the fermented seed is used as a seasoning agent in food[10]. In traditional medicine practice, juice expressed from the stem bark is applied on open wounds as an astringent and to cleanse the wound surface. The bark is also crushed to a pulp and placed on the wound surface as a dressing. Due to its use in wound treatment in traditional medicine, we evaluated the potentials of this plant as wound care agent by studying the effects of the stem bark extract on different parameters of wound care.

## MATERIALS AND METHODS

Adult Swiss albino rats (150-200 g) and mice (19-22 g) of either sex bred in the laboratory animal facility of the Department of Pharmacology and Toxicology, University of Nigeria, Nsukka were used for the study. The animals were freely maintained on standard pellets and water. All animal experiments were in compliance with the National Institute of Health Guide for Care and Use of Laboratory Animals (Pub No. 85-23, revised 1985). Neomycin-bacitracin powder (Cicatrin®, GlaxoSmithkline, USA) was purchased from a Pharmacy while dimethylsulphoxide (DMSO) and methanol (Sigma Aldrich, Germany) were purchased from the dealers.

### Preparation of extract:

Fresh stem bark of *P. africana* was collected in May, 2007 from Edem-Ani, Enugu State, Nigeria. The plant material was identified and authenticated at the International Centre for Drug Development (InterCEDD) Nsukka, Enugu State, Nigeria, where a voucher specimen is deposited (specimen number: INTERCEDD\508). The stem bark was cut into small pieces, dried under the sun and reduced to coarse powder using a mechanical blender. The powdered plant material (600 g) was extracted with methanol (100%) by cold maceration in a closed vessel with intermittent shaking for 48 h. The extract was filtered and concentrated in a rotary evaporator under reduced pressure (40-50°) to obtain 81.6 g (13.6% w/w) of the methanol extract (*P. africana* methanol extract; PAME) which was subsequently subjected to phytochemical analysis using standard procedures[11,12].

### Acute toxicity and lethality (LD_50_) tests:

The acute toxicity and lethality of PAME was determined in mice via the intraperitoneal route using the method described by Lorke (1983)[13]. Nine mice randomly divided into three groups (n=3) received 10, 100, and 1000 mg/kg of PAME in distilled water and were observed for 24 h for death. All the animals that received 1000 mg/kg were died. Hence, 140, 225, 370 and 600 mg/kg were administered respectively to a fresh batch of animals (n=1). After 24 h, none of the animals died. Thus, the LD_50_ was calculated as the geometric mean of the highest non-lethal dose (600 mg/kg) and the lowest lethal dose (1000 mg/kg).

### Bleeding time in rats:

Adult Swiss albino rats of both sexes were randomly divided into five groups (n=5). The tail of each rat was cut with a sharp pair of scissors and immediately, a drop of the test substance was applied on the cut simultaneously with the start of the stopwatch. Each group received one drop (0.01 ml) of one of 0.1, 10 and 100 mg/ml of PAME. The control group received either the vehicle (distilled water) or normal saline. The cut was dabbed with a small piece of filter paper every 15 s until the paper no longer stained red with blood oozing from the cut. Bleeding time was taken as the time for the first drop of blood to show to the time when the filter paper stopped showing blood stain[6,14].

### Coagulation time of whole rat blood:

Adult Swiss albino rats of both sexes were randomly divided into five groups (n=5). Each animal was anaesthesized with ether and the thoracic cavity opened to expose the aorta. The aorta was severed and 1 ml of blood quickly withdrawn using a plastic disposable syringe and transferred into clean, uniform sized paraffin coated plastic tubes (2.5 cm diameter) containing 0.5 ml of one of 10, 50 and 100 μg/ml of PAME in distilled water[6,14]. The vehicle and normal saline served as control. The plastic tubes were swirled every 15 s to check the fluidity of the contents. The interval between the introduction of the blood and the time of clot formation was taken as the coagulation time[14].

### Excision wound:

The excision wound model is usually employed to study the rate of wound contraction and epithelialization. Briefly, twenty five rats were randomly divided into five groups (n=5). The animals were anaesthesized with ether and held in a standard crouch position. A circular seal of 2.5 cm uniform diameter was impressed on the shaved dorsal thoracic central region, and the entire thickness of the skin from the marked area excised[15] to obtain a wound of about 500 mm^2^. Groups I-V were topically treated with distilled water (0.2 ml/kg), neomycin-bacitracin powder, PAME (100 or 200 mg/kg) and a combination of PAME (200 mg/kg) and neomycin-bacitracin powder respectively. Group V animals had their wounds cleaned with cotton wool soaked with PAME (200 mg/kg), followed by treatment with neomycin-bacitracin powder. The animals were housed individually in metal cages and treated once daily from day zero till the wound healed or up to day 21 post wounding, whichever was earlier. The wound area was measured on alternate days and compared with the wound area on day zero. The level of wound contraction (%) was calculated using the relation; wound contraction $(\;\%\;)\;=\;[(W_{\mathrm{Ao}}–W_{\mathrm{AT}})/(W_{\mathrm{AO}})]\times 100$ , where W_AO_ = wound area on day 0; W_AT_ = wound area on day t. Epithelialization time was measured as the time for fall of eschar leaving no raw wound[15,16].

### Dead space wound:

Ten adult Swiss albino rats were randomly placed in two groups (n=5). Two sterilized cotton pellets (30 mg each) were implanted subcutaneously under ether anaesthesia, one on either side in the lumbar region on the dorsal surface of each rat[17]. Group I received PAME (400 mg/kg) while group II received the vehicle (1 ml/kg) daily via the oral route from day 0 to 9 post wounding. The wounds were sutured with silk and mopped with an alcoholic swab and the animals placed in their individual cages. On day 10, the implanted cotton pellets with the granuloma tissues were carefully excised from the dead space under ether anaesthesia. The granuloma tissues obtained were dried in an oven at 60° to a constant weight and the weight recorded. The level of increase (%) in the weight of granuloma tissue formed was calculated relative to the control.

### Antimicrobial activity test:

The antibacterial effect of PAME was evaluated using laboratory strains of *Staphylococcus aureus, Klebsiella pneumoniae, Salmonella typhi, Pseudomonas aeruginosa* and *Bacillus subtilis* using the agar well diffusion method of Lovian (1980)[18]. Briefly, sterile Muller Hinton agar plates were flooded with 1×10^6^ cfu/ml concentration of the microorganisms. Using a sterile cork borer (7 mm diameter), 6 wells were bored on the agar and 3 drops of different concentrations of PAME (100, 50, 25, 12.5, 6.25 mg/ml) dissolved in 10% dimethylsulfoxide (DMSO) in water placed in the appropriate well. The DMSO (10%) was used as vehicle control, while gentamicin (1.4 mg/ml) served as standard. The plates were allowed 30 min for diffusion and incubated in an inverted position for 24 h at 37°. Microbial sensitivity was determined in triplicate. After incubation, the inhibition zone diameter (IZD) for each well was measured horizontally and vertically and the mean obtained. The minimum inhibitory concentration (MIC) was determined as intercept on the concentration axis of log concentration against mean IZD[2] plot.

### Statistical analysis:

Data was analyzed using One way Analysis of Variance (ANOVA) and the results expressed as Mean±SEM. Data was further subjected to LSD post hoc test for multiple comparisons and differences between means accepted significant at *p*<0.05.

## RESULTS AND DISCUSSION

Assessment of the effects of PAME on parameters of wound care revealed potent haemostatic and antibacterial activities with mild acceleration of wound healing. Studies on haemostatic activity showed significant (*p*<0.05) dose-related reduction in bleeding time in rats (Table 1) and coagulation time of whole rat blood *in vitro* (Table 2). Bleeding due to injury is a direct consequence of vascular damage and must be arrested by haemostatic mechanisms for healing and repair of the wounded tissue to commence. Haemostasis involves a cascade of reactions starting with vasospasm, platelet adhesion and aggregation to platelet plug formation and blood coagulation. The extent of interaction of PAME or its constituents with these mechanisms is not known. However, reduction in coagulation time suggests the haemostatic activity may derive, albeit in part, from acceleration of the coagulation process.

**TABLE 1:** EFFECT OF PAME ON BLEEDING TIME IN RATS

Treatment	Concentration (mg/ml)	Bleeding time (min)	Reduction in bleeding time (%)
PAME	0.1	8.81±1.1*	41.42
	10	8.74±1.4*	41.89
	100	5.74±1.5*	61.84
Normal saline	-	8.21±1.2*	45.41
Control	-	15.04±2.8*	-

*n* = 5;

* *p*<0.05 compared to control (One Way ANOVA; LSD post hoc); Reduction (%) in bleeding time was calculated relative to control.

**TABLE 2:** EFFECT OF PAME ON THE COAGULATION TIME OF WHOLE RAT BLOOD

Treatment	Dose (μg/ml)	Coagulation time (s)	Reduction in coagulation time (%)
PAME	10	19.20±1.9*	76.98
	50	25.00±3.4*	70.02
	100	11.20±1.2*	86.57
Normal saline	-	27.40±3.0*	67.15
Control	-	83.40±5.5*	-

*n*=5;

* *p*<0.05 compared to control (One Way ANOVA; LSD post hoc); Reduction (%) in coagulation time was calculated relative to control.

**Fig. 1:**
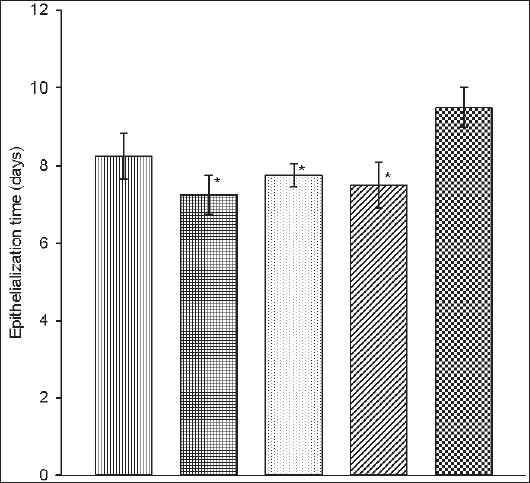
Effect of PAME on epithelialization time of excision wound 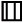 PAME (100 mg/kg), 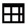 PAME (200 mg/kg), 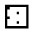 PAME+NEO-BAC, 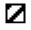 NEO-BAC and 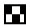 Control; n=5, *p<0.05 compared to control (One Way Anova; LSD post hoc); NEO-BAC=Neomycin-bacitracin

Studies on wound healing showed that PAME significantly (*p*<0.05) reduced epithelialization period of excision wounds in a dose-related manner. The effect was slightly better than that of neomycin-bacitracin and PAME-neomycin-bacitracin combination and occurred in the order of magnitude PAME>neomycin-bacitracin>PAME-neomycin-bacitracin (fig. 1). The PAME also caused little or no increase in the contraction rate of excision wounds with no significant difference among treatment groups (Table 3). Epithelialization or renewal of epithelial tissues after injury involves the proliferation and migration of epithelial cells towards the centre of the wound while wound contraction is largely due to the action of myofibroblasts[19,20]. This suggests that PAME may enhance the migration and proliferation of epithelial cells with little or no effect on myofibroblasts principally responsible for contraction. This type of differential action in wound healing is not unusual[21,22] as the two phases run independently[20,23]. Further evaluation showed that PAME mildly enhanced granuloma tissue formation in dead space wounds. The PAME caused a slight increase (94.75±5.4 mg; 15%) in granuloma tissue weight compared to control (82.40±11.0 mg). Granuloma tissue formed on an inert foreign body in a dead space comprises an accumulation of modified macrophages[5] and histological giant cells and undifferentiated connective tissue consisting largely of collagen[5,20,23]. Increase in granuloma tissue in dead space wound is associated with enhanced collagen maturation and increased protein content as well as angiogenesis[24–26] in the wound. These processes are indicators of generation of new tissues and suggest that PAME may stimulate mechanisms associated with tissue regeneration. Consistent with this is the effect of growth factors secreted by macrophages on wounds. Macrophages are a recognized source of major peptide growth factors that exert pro-healing effect by stimulating regeneration, fibroblast proliferation and activation, and angiogenesis[5]. Therefore, it is likely that in addition to enhancing epithelial cell migration, PAME may directly stimulate tissue regeneration processes and amplify same indirectly, through the action of tissue growth peptides secreted by macrophages whose accumulation it enhances.

**TABLE 3:** EFFECT OF PAME ON EXCISION WOUNDS IN RATS

Treatment	Dose (mg/kg)	Wound contraction (%)							
		Day 1	Day 3	Day 5	Day 7	Day 9	Day 11	Day 13	Day 15
PAME	100	3.14±0.50	6.16±1.08	21.51±9.11	35.96 ±9.12	73.83±4.06	91.38±1.27	93.28±0.68	96.46±0.82
	200	8.68±2.23	13.01±2.81	27.97±5.57	46.43±10.85	76.81±2.28	88.29±2.74	94.73±1.25	95.69±1.03
PAME-NEO-BAC	200	11.84±4.91	17.11±4.20	29.38±2.61	58.29±4.10	72.82±1.40	90.84±1.99	93.53±1.90	96.14±0.63
NEO-BAC	-	9.38±2.73	19.86±6.65	35.17±4.69	56.71±6.23	81.96±3.73	88.77±2.19	93.69±1.19	95.05±1.69
Control	-	8.07±4.29	9.97±4.77	24.72±1.09	56.67±4.69	70.57±7.08	88.44±2.40	92.54±1.43	94.61±0.45

*n*= 5; Wound contraction (%) was calculated relative to wound diameter on Day 0; NEO-BAC = Neomycin-bacitracin.

Despite the deployment of appropriately enhanced tissue growth and repair processes, microbial contamination poses a great threat to timely and successful healing of wounds. Unfortunately, wounds provide environment conducive for the growth of microbial organisms. Microbial infection of wounds delays healing[20,27,28] and causes a more pronounced acute inflammatory reaction[5] which can lead to further tissue injury and damage. Several organisms such as *Pseudomonas aeruginosa, Staphylococcus aureus, Streptococcus faecalis, Escherichia coli, Clostridium perfringens, Clostridium tetani, Coliform bacilli* and *Enterococcus*[28,29] have been implicated as wound contaminants. Evaluation of the antimicrobial activity showed that PAME inhibited the growth of cultures of *S. aureus, B. subtilis, S. typhi, P. aeruginosa* and *K. pneumoniae* to varying extents. The magnitude of sensitivity of the bacterial strains and hence antibacterial effect is of the order *B. subtilis > S. aureus > S. typhi > P.aeruginosa > K. pneumoniae* as shown by the minimum inhibitory concentrations (Table 4). Although these organisms were not wound isolates, inhibition of their growth indicates that PAME may protect wounds from contamination or infection with these and related microbes. This would enhance wound healing by allowing the natural tissue growth and repair processes already activated at the time of wound creation to proceed unhindered. Elsewhere, antimicrobial activity has been shown to contribute to the wound healing effects of honey, essential oil of *Melaleuca alternifolia* and leaf extracts of *Aspilia africana*[6,28,30,31]. It is thus likely, that the antibacterial activity of PAME may contribute to its wound healing potential. The PAME may also provide the additional benefit of accelerating the healing of contaminated wounds by eradicating already established infection by susceptible microorganisms.

**TABLE 4:** ANTIBACTERIAL ACTIVITY OF PAME

Treatment	Minimum inhibitory concentration (mg/ml)				
	*S. aureus*	*B. subtilis*	*S. typhi*	*P. aeruginosa*	*K. pneumoniae*
PAME	5.43	4.30	7.89	8.83	10.27
Gentamicin	0.04	0.02	0.36	0.40	0.06

Phytochemical analysis revealed the presence of alkaloids, carbohydrates, glycosides, tannins, reducing sugar, steroids, terpenoids, flavonoids and saponins which are typical plant constituents responsible for the diverse pharmacological activities of medicinal plants. These constituents may act in concert to mediate the effects of PAME in wound care. The abundance of tannins, which has been implicated in the haemostatic activity of plants where they arrest bleeding from damaged or injured vessels by precipitating proteins to form vascular plugs, may partly contribute to the haemostatic activity of PAME.

Toxicological studies for acute toxicity and lethality established an intraperitoneal LD_50_ of 774 mg/kg in mice. This suggests PAME possesses some degree of relative safety from acute intoxication.

In conclusion, the results of this study showed that the stem bark of *P. africana* has potentials for use in wound care due to the ability of its constituents to arrest bleeding from fresh wounds, inhibit the growth of bacterial wound contaminants and accelerate wound healing by enhancing epithelialization and stimulating tissue proliferation/re-modelling.
